# Treatment results of high dose cabergoline as an adjuvant therapy in six patients with established severe ovarian hyper stimulation syndrome

**Published:** 2014-10

**Authors:** Nasrin Saharkhiz, Azadeh Akbari Sene, Saghar Salehpour, Maryam Tamimi, Masoumeh Vasheghani Farahani, Kourosh Sheibani

**Affiliations:** 1 *Infertility and Reproductive Health Research Center (IRHRC), Shahid Beheshti University of Medical Sciences, Tehran, Iran.*; 2 *Iran University of medical Sciences, Shahid Akbar-abadi HospitalIVF Center, Tehran, Iran.*; 3 *Department of Obstetrics and Gynecology, Shahid Beheshti University of Medical Sciences, Tehran, Iran.*; 4 *Research and Development Center, Imam Hossein Hospital, Shahid Beheshti University of Medical Sciences, Tehran, Iran.*

**Keywords:** *Ovarianhyperstimulationsyndrome*, *Cabergoline*, *Paracentesis*, *Ovulationinduction*, *Invitrofertilization*

## Abstract

**Background:** The beneficial role of cabergoline as a prophylactic agent to prevent ovarian hyper stimulation syndrome (OHSS) among high-risk patients has been demonstrated in previous studies. But data for its role as a treatment for established severe OHSS is still limited. We represent the treatment results of high dose oral cabergoline in management of six patients after the syndrome is established.

**Case: **High-dose oral cabergoline (1 mg daily for eight days) was prescribed as an adjuvant to symptomatic treatment for six hospitalized patients with established severe OHSS following infertility treatment cycles. In two cases OHSS resolved rapidly despite the occurrence of ongoing pregnancy.

**Conclusion:** Considering the treatment outcomes of our patients, high dose cabergoline did not eliminate the need for traditional treatments, but it was a relatively effective and safe therapy in management of established severe OHSS, and prevented the increase in its severity following the occurrence of pregnancy.

## Introduction

Ovarian Hyperstimulation Syndrome (OHSS) is a potentially life threatening condition which is characterized by enlarged ovaries and fluid shift to the third-space and mostly happens following controlled ovarian hyperstimulation during assisted reproduction treatment (1). Although OHSS is a dangerous condition all established treatments are symptomatic and there is no treatment available based on the pathophysiology of this condition, so there is a need for better treatment methods. It seems that increased vascular permeability is the main pathophysiological aspect related to OHSS.

In women undergoing ovulation induction there is a surge in production of vascular endothelial growth factor (VEGF) in ovarian follicles during stimulation period, which is intensified following HCG injection. VEGF increases the vascular permeability (VP) upon binding to its type 2 receptors (VEGFR2) (2). It is demonstrated that Dopamine administration causes a reduction of phosphorylation of VEGFR2 leading to decreased permeability of VEGF dependent vasculature (3). Several studies have reported the use of cabergoline (a dopamin agonist) as a prophylactic treatment for OHSS among high-risk patients (4, 5).

Although in some recent studies the role of cabergoline as an effective prophylactic agent to reduce the chance of OHSS occurrence in high risk patients has been demonstrated; but there is limited knowledge about its effectiveness in treatment of patients with established OHSS, and not just preventing the condition as well as its optimum dose for treatment, have not been established (6). In one case report, increasing the dose of cabergoline to 1 mg per day in a patient who showed severe OHSS after receiving the prophylactic daily dose of 0.5 mg of drug showed good treatment results (7). In the present study we used this same treatment protocol of high dose cabergoline and studied the effectiveness of treatment with 1 mg of drug in six patients with established severe OHSS.

## Case report

In the present case series, we report the treatment results of six infertile patients with established severe OHSS- based on Navot classification- referred to our center from 2010 to 2011, who were treated with high dose oral cabergoline (8). According to the Navot classification, all patients with severe OHSS had variably enlarged ovaries, massive ascites with or without hydrothorax, a hematocrit greater than 45%, a leukocyte count greater than 15000, clinically measured oliguria, serum creatinine 1.0-1.5, laboratory evidence of hepatic dysfunction, and anasarca (8). 

The average age of our patients was 25.50±1.60 years. None of the patients were in critical condition based on Navot classification and all patients had BMI in normal range (8). The cause of infertility, infertility treatment method, the stimulation protocol, ovulation induction trigger and the onset of OHSS manifestation are reported in table I. Also all patients gave written informed consent before entering the study. We prescribed oral cabergoline (Dostinex 0.5 mg, Pfizer, Italy), 1 mg daily for all patients during an eight days period starting from the day of admittance. 

All patients also received prophylactic anticoagulant therapy and intravenous albumin for oliguria (average 8.67±2.67 of 20% vials for each patient). Also two of our patients underwent sonography guided transvaginal ascites paracentesis due to persistent oliguria, abdominal distention and discomfort, or shortness of breath (on day 4 and 7 after admission and Dostinex initiation for patient number1 and on day 5 for patient number 5). Patients were released from hospital when they showed progressive reduction of abdominal circumference and weight, and their CBC, electrolytes, liver function tests, renal function tests and coagulation tests were all normal. 

The shortest hospitalization time was 3 days for patient number 4 and the longest hospital stay was 9 days for patient number 1. In patients undergoing in vitro fertilization (IVF), embryos were frozen for future transfer but among 4 patients undergoing intra uterine insemination (IUI) or only induction of ovulation procedure (IO) followed by timed intercourse one resulted in term pregnancy with healthy neonate and the other one resulted in twin pregnancy with two healthy babies delivered at 37^th^ week of pregnancy (Table I). We did not observe any side effects for 1mg daily dose of Dostinex in any patient. 

**Table I:** Characteristics of 6 patients, their infertility treatment, stimulation protocols and fertility outcomes

**Case**	**Age**	**Etiology of infertility**	**Treatment cycle**	**Stimulation**	**Trigger of ovulation**	**Onset of OHSS**	**Pregnancy outcome**
1	25	PCOS/ Male	IVF	SLP + Merional	HCG (10.000 u)	Early	Embryo Freeze
2	29	PCOS	IO	Clomiphene + Fostimon*	HCG (5.000 u)	Early	Term twin pregnancy
3	26	PCOS	IUI	Clomiphene + Fostimon*	Decapeptil (0.1 mg)	Late	Term single tone pregnancy
4	19	PCOS	IO	Clomiphene + Merional*	HCG (5.000 u)	Early	Negative
5	30	PCOS /Male	IVF	SLP + Menopure	HCG (5.000 u)	Early	Embryo Freeze
6	24	Male	IO	Clomiphene + Merional*	HCG (10.000 u)	Early	Negative

SLP: Standard long protocol

PCOS: Polycystic Ovarian Syndrome

IVF: In Vitro Fertilization

IUI: Intra Uterine Insemination

IO: Induction of Ovulation followed by timed intercourse

* Sequential stimulation

## Discussion

Although OHSS is one of the most serious side effects of controlled ovarian hyperstimulation among infertile women, traditional treatments are all symptomatic and nonspecific. Considering the fact that the relation between VEGF and VP during OHSS in animal studies has been well established, one can articulate that a treatment based on pathophysiologic aspects should target VEGF-VEGFR2 system (9, 10). Gomez *et al* have indicated in their studies that VP is increased after hCG administration in super ovulated animals and there is a narrow correlation between ovarian mRNA VEGF expression and VP. They also reported an increase of VEGFR-2 expression in ovaries coincidental in time with maximal VP, demonstrating the involvement of VEGF-VEGFR-2 system in development of OHSS (3, 9, 10). It has been shown that dopamine receptor 2 (Dp-r2) agonists inhibit VEGFR-2-dependent VP and angiogenesis when administered at high doses in animal cancer models (5). 

To test whether VEGFR-2-dependent VP and angiogenesis could be segregated in a dose-dependent fashion with the Dp-r2 agonist cabergoline, Gomez *et al* used a well-established OHSS rat model supplemented with prolactin. In a study by Gomez *et al* in 2006 the authors reported that a 100 μg/kg low-dose of cabergoline reversed VEGFR2-dependent VP, without affecting luteal angiogenesis by partial inhibition of ovarian VEGFR-2 phosphorylation (5). In the same study no luteolytic effects on serum progesterone levels and luteal apoptosis were observed (5).

Early studies among egg donors and patients in risk of OHSS showed good results with a 0.5 mg cabergoline daily dose starting from the day of hCG administration (11, 12). This reduction was reported to be 20% among women at high risk of OHSS (11). In another study the incidence of OHSS was reduced among women with polycystic ovaries who were under cabergoline treatment due to hyperprolactinemaia during ovulation induction (13). Also in a recent meta-analysis by Tang *et al* the researchers concluded that the usage of cabergolin appears to reduce the risk of OHSS in high-risk women, particularly for moderate OHSS and has no adverse effect on pregnancy outcome (the clinical pregnancy rate and abortion rate), and there is no increased risk of adverse events (6).

Few other non-controlled clinical trials have studied the effect of dopamine agonists in treatment of established moderate to severe OHSS. They have demonstrated an improvement of urine output as well as reduction of ascites among patients with OHSS after administration of oral docarpamine (dopamine prodrug) or intravenous dopamine injection (14, 15). Rollene *et al *have reported good results treating four patients with moderate to severe OHSS using GnRH antagonist and a daily dose of 0.5mg cabergoline (16).

Ata *et al* have reported a patient who despite treatment with prophylactic 0.5 mg daily dose of cabergoline starting from the day of hCG administration developed moderate OHSS two days after oocyte retrieval. The investigators increased the dose to 1mg daily and continued the treatment for two weeks. Despite the occurrence of pregnancy OHSS symptoms subsided in this patient and a term and healthy newborn was delivered. The investigators concluded that the higher cabergoline dose might have prevented an increase in the severity of OHSS and its prolongation following occurrence of pregnancy (7). As Ata *et al *have hypothesized, the rationale behind increasing the daily Dostinex dose in our study was to prevent the progression of the already established syndrome by further decreasing the number of VEGFR2 available to stimulation by the VEGF molecules in the circulation (7).

Among our patients a daily dose of 1mg cabergoline did not cause a total independence of patients on supportive therapies such as volume expanders and paracenthesis in some cases; but it seems that a high dose of cabergoline beside other supportive measures can stop the progression of OHSS and stop the organ damage. In two of our patients despite the pregnancy (twin pregnancy in one patient), which is known to worsen the severity and prolong the OHSS, the symptoms subsided and a relatively fast recovery was achieved. It has been demonstrated that a 0.5mg daily dose of cabergoline does not impact the implantation and pregnancy among patients undergoing IVF (17). In the present study we did not observe any adverse impact after treatment with a daily dose of 1mg cabergoline for eight days on the maternal or fetal outcome if the pregnancy occurred. 

In recent years there has been some concern about the chance of heart valve diseases caused by long term usage of high dose dopaminergic drugs. But in multicentre study by Halperin *et al* except for an increase in mild tricuspid valve regurgitation with high cumulative dosage of cabernagoline (>180 mg) no increase in rate of other valve diseases was observed in hyperprolactinemic patients (18). Also in another study the researchers did not detect any significant valvular thickening or regurgitation after performing transthoracic echocardiography on 50 prolactinoma patients. These patients all received cumulative doses of 443±53 mg cabergoline for 6.6±0.5 years (19). 

Considering our results it seems that high dose cabergoline, as the only treatment based on pathophysiological aspects, could act effectively to diminish the clinical symptoms of established severe OHSS and prevent its prolongation in the case of pregnancy. Considering the fact that the daily dose of 0.5mg cabergoline has been chosen based on animal study results and is the average dosage used for treatment of hyperprolactinemia, more randomized controlled clinical trials with larger study population seems necessary to establish the optimum dosage and length of medication for prevention and treatment of OHSS (11).

Considering the outcome of patients in the present study using a daily cabergoline dose of 1mg did not eliminate the need for traditional treatments, but the use of 1mg cabergoline seemed to be a safe and effective adjacent therapy in established severe OHSS cases even with the occurrence of pregnancy in our limited number of patients. It has to be emphasized that further RCTs with large number of patients are required to assess the safety and effectiveness of this protocol on OHSS patients and on fetal outcome.

Also researchers should conduct more studies, including large RCTs to compare cabergoline and established treatments like interavenous albumin and paracentesis, to find the probable role of this drug as a treatment for established severe OHSS. Motta *et al* in their study on patients with intolerance to dopaminergic drugs found that the vaginal use of cabergoline (0.5 mg 2-5 times per week) is a safe and effective method to treat hyperprolactinemia and will eliminate the side effects of oral prescription (20). The nausea and vomiting caused by oral cabergoline might be added to the nausea and vomiting caused by ovarian stimulation might cause insufficient absorption, so we recommend further research on vaginal usage of cabergoline in OHSS patients. 
